# Hsa_circ_0005567 Activates Autophagy and Suppresses IL-1β-Induced Chondrocyte Apoptosis by Regulating miR-495

**DOI:** 10.3389/fmolb.2020.00216

**Published:** 2020-08-25

**Authors:** Jinling Zhang, Fangyue Cheng, Genxiang Rong, Zhi Tang, Binjie Gui

**Affiliations:** ^1^Department of Orthopedic, The First Affiliated Hospital of Anhui Medical University, Hefei, China; ^2^Department of Rheumatology, The First Affiliated Hospital of Anhui Medical University, Hefei, China

**Keywords:** circ_0005567, chondrocyte apoptosis, autophagy, miR-495, ATG14

## Abstract

Excessive chondrocyte apoptosis is mostly responsible for the progression of osteoarthritis (OA). It has been shown that circular RNAs (circRNAs) are differentially expressed in OA cartilage and participate in various pathological processes during OA. Here, this study was designed to explore the effect and molecular mechanism of hsa_circ_0005567 on IL-1β-induced chondrocyte apoptosis. The results showed that hsa_circ_0005567 knockdown aggravated the IL-1β-induced chondrocyte apoptosis. In contrast, hsa_circ_0005567 overexpression attenuated the IL-1β-induced chondrocyte apoptosis, but this effect could be abrogated by 3-methyladenine (an inhibitor of autophagy), suggesting that hsa_circ_0005567 overexpression inhibited chondrocyte apoptosis by inducing autophagy. Furthermore, hsa_circ_0005567 competitively bound to miR-495 and derepressed the expression of ATG14, an early autophagy marker that was a direct target of miR-495. Moreover, both miR-495 mimic and ATG14 knockdown counteracted the autophagy-promoting and anti-apoptotic effects of hsa_circ_0005567 overexpression in IL-1β-treated chondrocytes. Taken together, hsa_circ_0005567 activates autophagy by regulating the miR-495/ATG14 axis and thereby suppresses IL-1β-induced chondrocyte apoptosis. These findings suggest that hsa_circ_0005567 may serve as a therapeutic target for the treatment of OA.

## Introduction

Osteoarthritis (OA) is a degenerative disease with clinical manifestations such as joint pain, restricted mobility, and joint deformities (Yu et al., 2018). OA mainly occurs in the articular cartilage that is composed of chondrocytes and extracellular matrix (ECM). Chondrocytes are responsible for the secretion and transformation of ECM (Saitou et al., 2018). Excessive chondrocyte apoptosis and ECM degradation are associated with biological and functional degeneration in OA (Hwang and Kim, 2015; Lü et al., 2020). Interleukin-1β (IL-1β), one of the important pro-inflammatory cytokines abundantly expressed in OA patients, has been demonstrated to accelerate apoptosis of chondrocytes and mediate impairment of ECM deposition (Chen et al., 2018; Vincent, 2019; Frischholz et al., 2020). Inhibition of chondrocyte apoptosis is an important therapeutic target for OA therapy.

Circular RNAs (circRNAs) are a novel kind of long non-coding RNAs, which are characterized by a covalently closed continuous loop without 5′ or 3′ polarities structure (Rong et al., 2017; Kristensen et al., 2019). CircRNAs have a variety of biological functions and play an important regulatory role in multiple diseases (Shafabakhsh et al., 2019; Yin et al., 2019), including OA (Li et al., 2018). Increasing evidence shows that circRNAs are differentially expressed in OA cartilage and participate in various pathological processes during OA (Yu and Sun, 2018). For example, circRNA-CER could promote ECM degradation by acting as a sponge of miR-316 to upregulate the expression of metalloproteinase (MMP)-13 (Liu et al., 2016). CircSERPINE2 could competitively bind to miR-1271-5p to suppress chondrocyte apoptosis and ECM degradation (Shen et al., 2019). However, the function of circRNAs in OA remains largely unknown.

Xiao et al. (2019) identified the expression profile of circRNAs in OA knee condyle by illumina sequencing platform and found that a novel circRNA hsa_circ_0005567 (hereinafter referred to as “circ_0005567”) was downregulated in severe OA knee condyle, suggesting that circ_0005567 might participate in the occurrence and development of OA. The role of circ_0005567 in OA remains limited. Hence, this study was designed to explore the effect and molecular mechanism of circ_0005567 on IL-1β-induced chondrocyte apoptosis.

## Materials and Methods

### Cell Culture

Human primary chondrocytes were purchased from Procell (#CP-H107; Procell; Wuhan, China) and cultured in human chondrocyte complete medium (#CM-H107; Procell) at 37°C and 5% CO_2_. Recombinant human IL-1β (10 ng/mL) was added to stimulate degeneration of chondrocytes for 24 h. 3-methyladenine (3-MA; 10 mM; an autophagy inhibitor) was used to pretreat chondrocytes before transfection with circ_0005567 overexpression vector.

### Cell Transfection

The circ_0005567 overexpression vector, control empty vector, si-circ_0005567, si-ATG14, si-Ctrl, miR-495 mimics, miR-495 inhibitors, mimic negative control (NC), and inhibitor NC were purchased from GenePharma (Shanghai, China). The sequence of si-circ_0005567 was CCTTTTGTTGGCAATCTCT. The sequence of si-ATG14 is GCGGCGATTTCGTCTACTT. The sequence of si-Ctrl is TTCAATAAATTCTTGAGGTTT. Transfection was performed using Lipofectamine 3000 reagent (Invitrogen, United States) in chondrocytes. Chondrocytes were collected for subsequent experiments 48 h post-transfection.

### GFP-LC3 Fluorescence

Chondrocytes were plated into 24-well plates (2 × 10^3^ cells per well). When the cell confluence reached 80–90%, chondrocytes were transfected with the green fluorescent protein-microtubule associated protein 1 (MAP1) light chain 3 (GFP-LC3) plasmid using FuGENE HD^®^ Transfection Reagent (Promega, United States) and then cultured for another 24 h. The number of punctate LC3 in at least 30 cells was manually counted and the average of the number of punctate LC3 in a single cell was calculated.

### Cell Apoptosis Assay

A commercial Annexin V-FITC/PI Apoptosis Detection Kit (Sigma-Aldrich, United States) was used to assess cell apoptosis. Analyses were carried out using the FACScan flow cytometry (BD Biosciences, United States) equipped with the FlowJo 7.6 software. The percentage of apoptotic cells is indicated by the sum of the numerical value represented in the upper right (annexin and PI positive) and lower right quadrant (annexin positive/PI negative). The “cell apoptosis rate” was the average of apoptosis rates from four times of flow cytometry analysis.

### Real-Time Quantitative PCR (qRT-PCR)

Total RNA of chondrocytes was extracted using the TRIzol reagent and was reverse transcribed into cDNAs using the Reverse Transcription Kit (Takara, China). SYBR Premix Ex Taq™ (Takara) was used for amplification and relative quantification of cDNA. Relative quantitative PCRs for miRNAs were carried out with SYBR PrimeScript miRNA RT-PCR Kit (Takara). The 2^–ΔΔCt^ method was used to calculate fold changes. GAPDH (for circ_0005567 and ATG14) and U6 (for miR-495) were used as the internal controls for normalization. The primers were as follows: circ_0005567-Forward: 5′-TCC AGTCTGATCCTTTTGTTGG-3′; circ_0005567-Reverse: 5′-CT GTTTCTTGCTGTAGACGGCT-3′; ATG14-Forward: 5′-TGTA CCTGGTCAGTCCAAGCTC-3′; ATG14-Reverse: 5′-CAGGTC GGTTTCTTCATCGCTG-3′; miR-495-Forward: 5′-ACAAAC ATGGTGCACTTC-3′; miR-495-Reverse: 5′-GAACATGTCTGC GTATCTC-3′; GAPDH-Forward: 5′-GTCTCCTCTGACTTCA ACAGCG-3′; GAPDH-Reverse: 5′-ACCACCCTGTTGCTGT AGCCAA-3′; U6-Forward: 5′-TGCGGGTGCTCGCTTCGCA GC-3′; U6-Reverse: 5′-CCAGTGCAGGGTCCGAGGT-3′.

### Western Blot

Western blot was performed as previously described (Chen et al., 2018). The primary antibodies were as follows: Beclin-1 (1:1,000; #ab62557; Abcam, United States), LC3-I and LC3-II (both from anti-LC3B antibodies, 1:1,000; #ab48394; Abcam), ATG14 (1:1,000; #5504; Cell Signaling Technology, United States), Caspase-3 (1:1,000; #ab32351; Abcam), Bax (1:500; #sc-7480; Santa Cruz Biotechnology, United States), Bcl-2 (1:500; #sc-7382; Santa Cruz Biotechnology), and β-actin (1:1000; #ab8227; Abcam).

### RNA Pull-Down Assay

Chondrocytes were lysed in 1 mL 0.1% NP40 lysate containing protease inhibitor. The biotin-labeled miR-495 or random pull-down probe sequence as NC was designed and synthesized. Biotinylated RNAs were then incubated with streptavidin agarose-treated magnetic beads and cell lysates for 1 h. The circ_0005567 expression precipitated by the biotin-labeled miR-495 was detected by qRT-PCR. Similarly, the miR-495 expression precipitated by the biotin-labeled circ_0005567 probe was also examined by qRT-PCR.

### Luciferase Reporter Assay

For reporter assays, chondrocytes at 70–80% confluency were co-transfected with 0.8 μg of reporter plasmids (pmirGLO-ATG14 WT or pmirGLO-ATG14 Mut) in the presence of 10 nM NC/miR-495 mimics using Lipofectamine 3000. The luciferase activity was measured 48 h later using the Dual-Luciferase Reporter Assay System (Promega).

### Statistical Analysis

SPSS 22.0 software was used for statistical analysis. The differences between two groups or multi-groups were analyzed by *t*-test and one-way ANOVA, respectively. *P* < 0.05 was considered statistically significant.

## Results

### Effect of Circ_0005567 Overexpression and Knockdown on the IL-1β-Induced Chondrocyte Apoptosis

**FIGURE 1:**
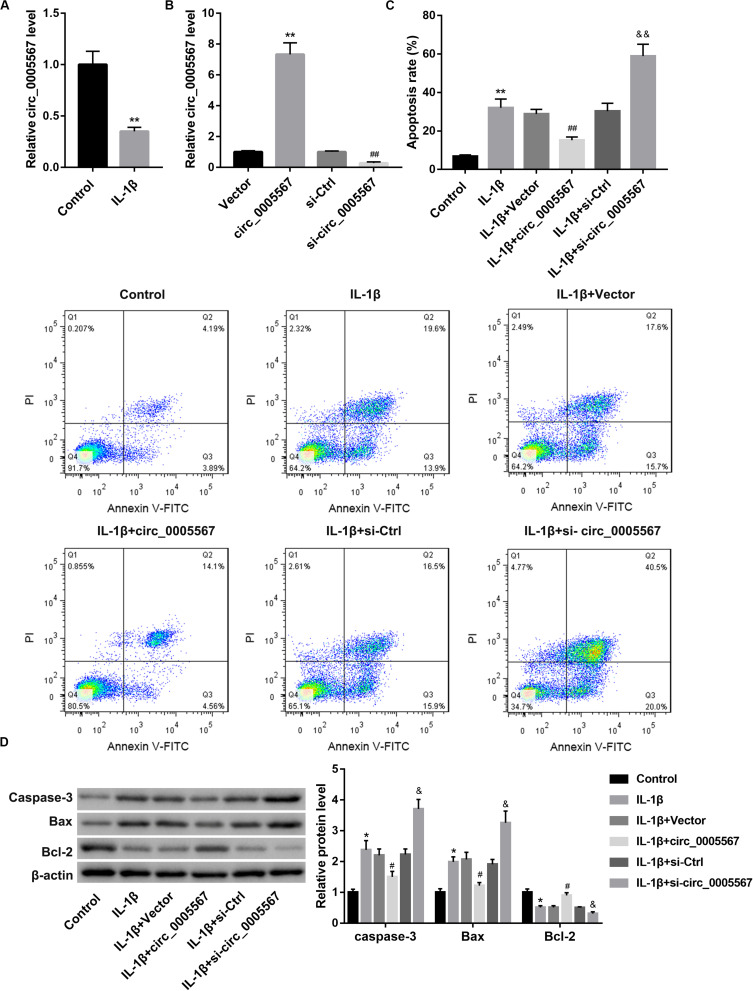
Effect of circ_0005567 overexpression and knockdown on IL-1β-induced chondrocyte apoptosis. **(A)** Circ_0005567 expression in PBS (control)- or IL-1β-treated chondrocytes was examined by qRT-PCR. **(B)** Circ_0005567 expression determined by qRT-PCR analysis, **(C)** cell apoptosis rate determined by flow cytometry after Annexin V-FITC/PI staining, and **(D)** protein levels of caspase-3, Bax, and Bcl-2 examined by western blot in chondrocytes transfected with circ_0005567 overexpression vector (circ_0005567 group), empty vector, si- circ_0005567, or si-Ctrl in the presence of PBS (control) or IL-1β. The data are expressed as mean ± standard deviation from three independent experiments. *P < 0.05, **P < 0.01, versus the Control or Vector group; ^#^*P* < 0.05, ^##^*P* < 0.01, versus the si-Ctrl or IL-1β + Vector group; ^&^*P* < 0.05, ^&&^*P* < 0.01, versus the IL-1β + si-Ctrl group.

IL-1β can stimulate degeneration of chondrocytes. We initially determined circ_0005567 expression in chondrocytes following 24 h of IL-1β stimulation. Circ_0005567 expression was significantly lower in the IL-1β-treated chondrocytes than that in the PBS-treated chondrocytes (Figure 1A). We then examined the effect of circ_0005567 overexpression and knockdown on IL-1β-induced chondrocyte apoptosis. qRT-PCR results confirmed the successful overexpression and knockdown of circ_0005567 in chondrocytes (Figure 1B). Cell apoptosis was evaluated using flow cytometry and western blot analysis of apoptosis-related proteins. Expectedly, IL-1β stimulation markedly increased cell apoptosis rate (Figure 1C) and protein levels of pro-apoptotic caspase-3 and Bax, but decreased protein level of anti-apoptotic Bcl-2 (Figure 1D). Of note, the IL-1β-induced chondrocyte apoptosis was counteracted by circ_0005567 overexpression but aggravated by circ_0005567 knockdown (Figures 1C,D). We also found that circ_0005567 silencing abrogated the inhibitory effects of circ_0005567 overexpression on cell apoptosis (Supplementary Figure S1A).

### Circ_0005567 Overexpression Attenuated the IL-1β-Induced Chondrocyte Apoptosis by Inducing Autophagy

**FIGURE 2:**
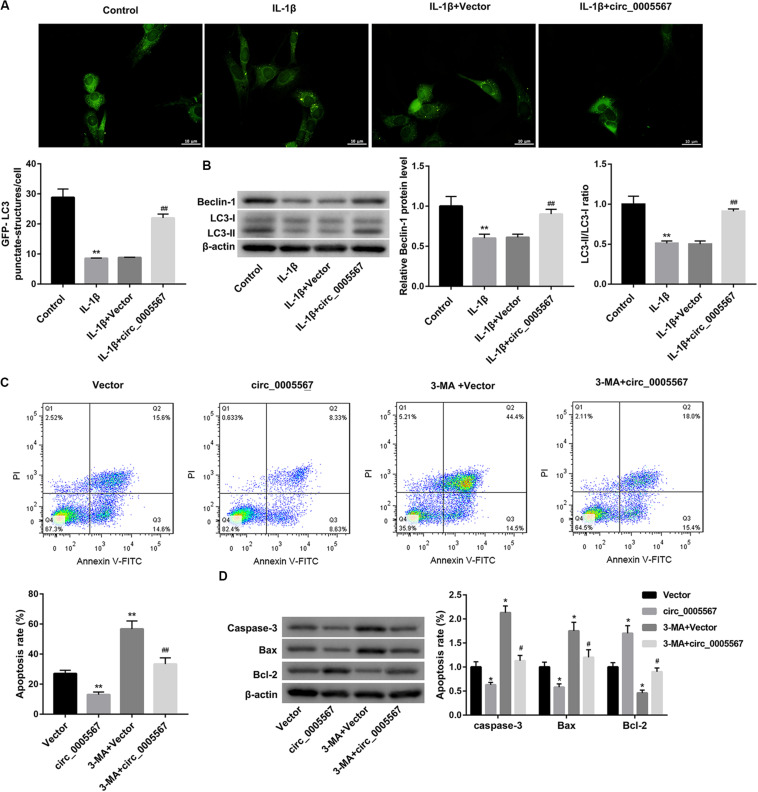
Circ_0005567 overexpression attenuated the IL-1β-induced chondrocyte apoptosis by inducing autophagy. **(A)** Immunofluorescence results of chondrocytes transfected with GFP-LC3 and **(B)** protein levels of Beclin-1 and LC3-II/LC3-I examined by western blot in chondrocytes transfected with circ_0005567 overexpression vector (circ_0005567 group) or empty vector in the presence of PBS (control) or IL-1β. **(C)** Cell apoptosis rate determined by flow cytometry after Annexin V-FITC/PI staining and **(D)** protein levels of caspase-3, Bax, and Bcl-2 examined by western blot in IL-1β-treated chondrocytes in the groups of Vector, circ_0005567, 3-MA + Vector, and 3-MA + circ_0005567. The data are expressed as mean ± standard deviation from three independent experiments. *P < 0.05, **P < 0.01, versus the Control or Vector group; ^#^*P* < 0.05, ^##^*P* < 0.01, versus the IL-1β + Vector or circ_0005567 group.

Activation of chondrocyte autophagy has been shown to exert a chondroprotective effect in OA (Feng et al., 2020). Thus, we sought to determine whether autophagy activation is involved in the protective effect of circ_0005567 in chondrocytes. Autophagy was assessed by GFP-LC3 immunofluorescence and western blot analysis of autophagy-related markers (LC3 and Beclin-1). The results showed that circ_0005567 overexpression restored the IL-1β-mediated inhibition of the number of GFP-LC3 punctate structures (Figure 2A) and protein levels of LC3-II and Beclin-1, as well as the ratio of LC3-II/LC3-I (Figure 2B). However, treatment with 3-methyladenine (3-MA), an inhibitor of autophagy, reversed the circ_0005567 overexpression-mediated promotion of autophagy (Supplementary Figure S2). Of note, 3-MA treatment abrogated the anti-apoptotic effect of circ_0005567 overexpression on IL-1β-stimulated chondrocytes (Figures 2C,D). These results implied that circ_0005567 overexpression attenuated the IL-1β-induced chondrocyte apoptosis by inducing autophagy.

### Circ_0005567 Derepressed ATG14 Expression by Sponging miR-495

**FIGURE 3:**
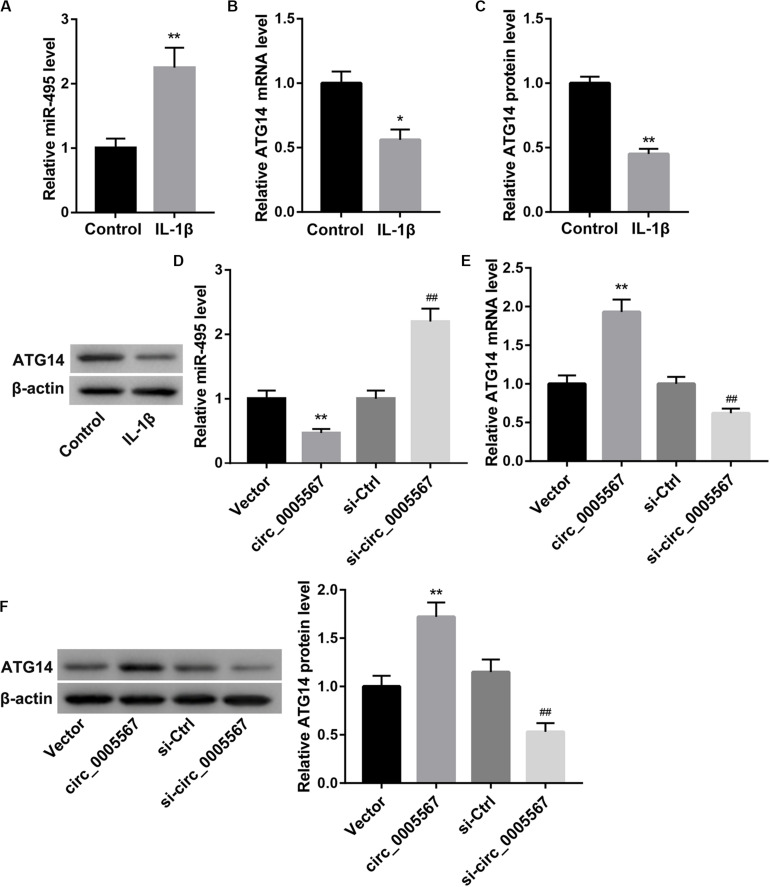
Circ_0005567 negatively regulated miR-495 expression but positively regulated ATG14 expression. **(A)** MiR-495 expression examined by qRT-PCR analysis, **(B)** ATG14 mRNA level determined by qRT-PCR analysis, and **(C)** ATG14 protein level examined by western blot in PBS (control)- or IL-1β-treated chondrocytes. **(D)** MiR-495 expression examined by qRT-PCR analysis, **(E)** ATG14 mRNA level determined by qRT-PCR analysis, and **(F)** ATG14 protein level examined by western blot in chondrocytes transfected with circ_0005567 overexpression vector, empty vector, si-circ_0005567, or si-Ctrl in the presence of IL-1β. The data are expressed as mean ± standard deviation from three independent experiments. *P < 0.05, **P < 0.01, versus the Control or Vector group; ^##^*P* < 0.01, versus the si-Ctrl group.

Our bioinformatics analysis revealed that there were putative binding sites between circ_0005567 and miR-495. ATG14, an early autophagy marker, was identified as a predictive target gene of miR-495. Thus, we speculated that circ_0005567 could competitively bind to miR-495 and relieve the targeted inhibition of ATG14 by miR-495, thereby promoting chondrocyte autophagy. IL-1β stimulation greatly increased miR-495 expression (Figure 3A), whereas decreased ATG14 mRNA and protein levels (Figures 3B,C) in chondrocytes. Furthermore, circ_0005567 overexpression resulted in a notable decrease in miR-495 expression but a significant increase in ATG14 mRNA and protein levels. On the contrary, circ_0005567 knockdown upregulated miR-495 expression, whereas downregulated that of ATG14 (Figures 3D–F). Furthermore, circ_0005567 silencing abolished the inducing effects of circ_0005567 overexpression on ATG14 mRNA level (Supplementary Figure S1B).

**FIGURE 4:**
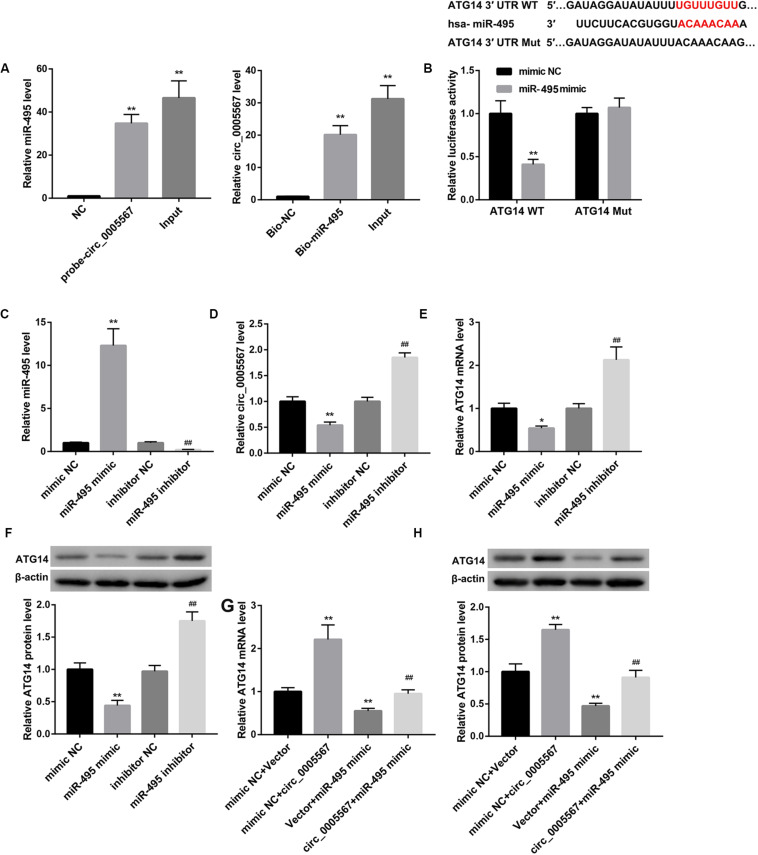
Circ_0005567 derepressed ATG14 expression by sponging miR-495. **(A)** RNA pull-down was performed to analyze the interaction between circ_0005567 and miR-495. **(B)** Sequences of the putative miR-495 binding sites in the 3′- UTR of ATG14. Luciferase activity in chondrocytes co-transfected with wild type (WT) or mutant (Mut) ATG14 3′-UTR luciferase reporter plasmids and miR-495 mimics or mimic NC. **(C)** MiR-495 expression, **(D)** circ_0005567 expression, **(E)** ATG14 mRNA level, and **(F)** ATG14 protein level in chondrocytes transfected with miR-495 mimic, mimic NC, miR-495 inhibitor, and inhibitor NC. **(G)** ATG14 mRNA level and **(H)** ATG14 protein level in chondrocytes co-transfected with miR-495 mimic/mimic NC and circ_0005567 overexpression vector/empty vector. The data are expressed as mean ± standard deviation from three independent experiments. *P < 0.05, **P < 0.01, versus the NC or mimic NC or mimic NC + Vector group; ^##^*P* < 0.01, versus the inhibitor NC or mimic NC + circ_0005567 or Vector + miR-495 mimic group.

Moreover, results from RNA-pull down experiments showed significant enrichment of miR-495 in the circ_0005567 pulled down pellet when compared with the NC group as measured using qRT-PCR. We also observed an enrichment of circ_0005567 in the miR-495 pulled down pellet. These data confirmed the direct interaction between circ_0005567 and miR-495 (Figure 4A). Luciferase reporter assay demonstrated that miR-495 mimic transfection notably reduced luciferase activity in the chondrocytes transfected with ATG14 WT reporter, suggesting that miR-495 directly targeted ATG14 3′-UTR (Figure 4B).

Also, miR-495 mimic transfection significantly upregulated miR-495 expression, whereas downregulated expression of circ_0005567 and ATG14. In contrast, miR-495 inhibitor transfection downregulated miR-495, whereas upregulated circ_0005567 and ATG14 expression (Figures 4C–F). The miR-495 mimic-mediated inhibition of ATG14 mRNA and protein levels was derepressed by circ_0005567 overexpression (Figures 4G,H). Collectively, these results manifested that circ_0005567 derepressed ATG14 expression by sponging miR-495.

### Circ_0005567 Promoted Autophagy and Inhibited Chondrocyte Apoptosis via miR-495/ATG14 Axis

**FIGURE 5:**
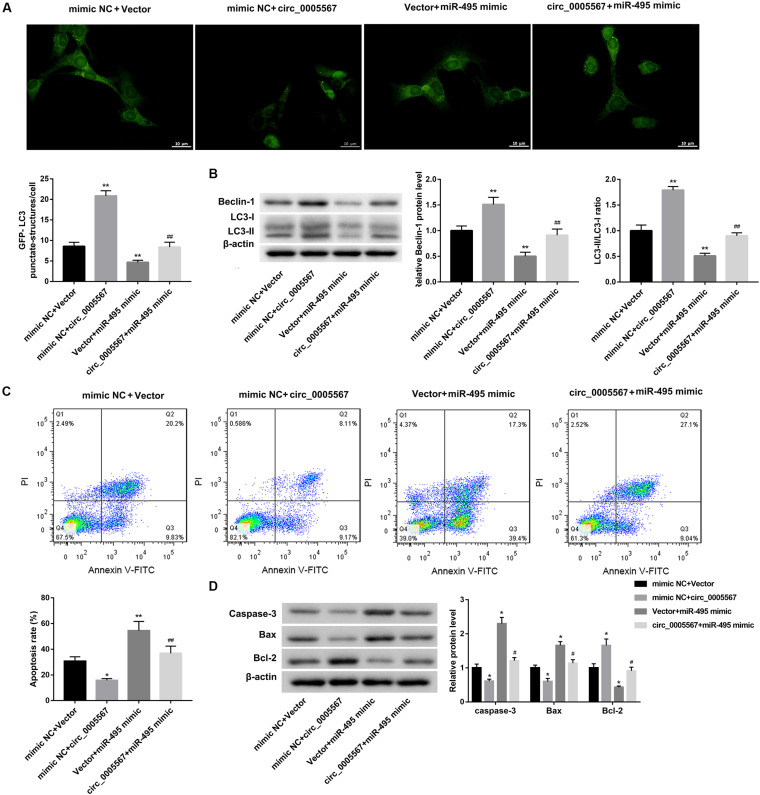
Circ_0005567 promoted autophagy and inhibited chondrocyte apoptosis by inhibiting miR-495 expression. **(A)** Immunofluorescence results of chondrocytes transfected with GFP-LC3, **(B)** protein levels of Beclin-1 and LC3II/LC3-I examined by western blot, **(C)** cell apoptosis rate determined by flow cytometry after Annexin V-FITC/PI staining, and **(D)** protein levels of caspase-3, Bax, and Bcl-2 examined by western blot in chondrocytes co-transfected with miR-495 mimic/mimic NC and circ_0005567 overexpression vector/empty vector in the presence of IL-1β. The data are expressed as mean ± standard deviation from three independent experiments. *P < 0.05, **P < 0.01, versus the mimic NC + Vector group; ^#^*P* < 0.05, ^##^*P* < 0.01, versus the mimic NC + circ_0005567 or Vector + miR-495 mimic group.

**FIGURE 6:**
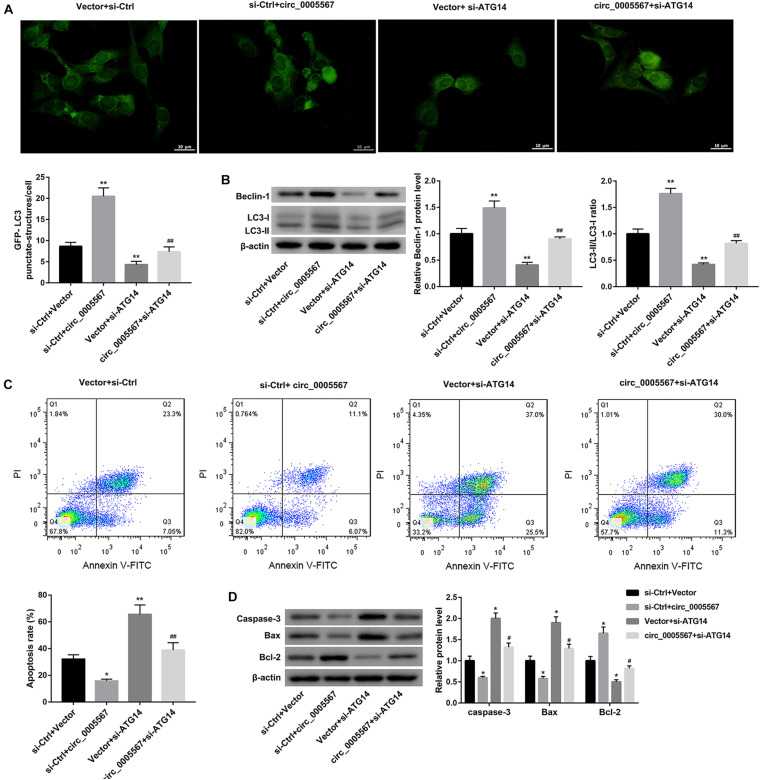
Circ_0005567 promoted autophagy and inhibited chondrocyte apoptosis by upregulating ATG14 expression. **(A)** Immunofluorescence results of chondrocytes transfected with GFP-LC3, **(B)** protein levels of Beclin-1 and LC3-II/LC3-I examined by western blot, **(C)** cell apoptosis rate determined by flow cytometry after Annexin V-FITC/PI staining, and **(D)** protein levels of caspase-3, Bax, and Bcl-2 examined by western blot in chondrocytes co-transfected with si-ATG14/si-Ctrl and circ_0005567 overexpression vector/empty vector in the presence of IL-1β. The data are expressed as mean ± standard deviation from three independent experiments. *P < 0.05, **P < 0.01, versus the si-Ctrl + Vector group; ^#^*P* < 0.05, ^##^*P* < 0.01, versus the si-Ctrl + circ_0005567 or Vector + si-ATG14 group.

Finally, we determined whether circ_0005567 promotes autophagy and inhibits chondrocyte apoptosis by sponging miR-495 to derepress ATG14 expression. MiR-495 mimic transfection significantly decreased the number of GFP-LC3 punctate structures (Figure 5A) and protein levels of LC3-II and Beclin-1, as well as the ratio of LC3-II/LC3-I (Figure 5B). Furthermore, the apoptosis rate and protein levels of pro-apoptotic proteins (caspase-3 and Bax) were notably upregulated, whereas the anti-apoptotic Bcl-2 protein level was significantly downregulated in IL-1β-treated chondrocytes following transfection with miR-495 mimic (Figures 5C,D). Importantly, miR-495 mimic transfection effectively abolished the circ_0005567 overexpression-mediated autophagy promotion (Figures 5A,B) and chondrocyte apoptosis inhibition (Figures 5C,D). Similarly, ATG14 knockdown could also abrogate the autophagy-promoting and anti-apoptotic effects of circ_0005567 overexpression in IL-1β-treated chondrocytes (Figure 6). Taken together, circ_0005567 promoted autophagy and inhibited chondrocyte apoptosis via the miR-495/ATG14 axis.

## Discussion

Circular RNAs play important roles in multiple diseases, including OA (Li et al., 2018; Yu and Sun, 2018). Inhibition of chondrocyte apoptosis is a valid therapeutic target for OA therapy. To the best of our knowledge, this present study provided the first evidence that circ_0005567 overexpression attenuated, whereas circ_0005567 knockdown aggravated the IL-1β-induced chondrocyte apoptosis. Thus, circ_0005567 may serve as a therapeutic target for the treatment of OA.

Autophagy is a major degradation pathway whereby cytosolic components are degraded within the lysosome (Yang and Klionsky, 2010). LC3-II is an indicator of autophagy because LC3 protein converts from LC3-I to LC3-II during autophagosome formation. Beclin-1 is also an important autophagy marker (Fîlfan et al., 2017). In this study, we assessed autophagy by measuring the expression of LC3 puncta, LC3-II, and Beclin-1. We here demonstrated for the first time that circ_0005567 promoted chondrocyte autophagy, as evidenced by increases in the number of GFP-LC3 punctate structures, the ratio of LC3-II/LC3-I, and Beclin-1 expression following circ_0005567 overexpression.

Autophagy exerts a protective role in chondrocytes and OA. The enhancement of autophagy in chondrocytes can delay the progression of OA (Luo et al., 2019). Treatment with the autophagy inhibitor 3-MA can aggravate the severity of experimental OA (Cheng et al., 2017). Zhou J. et al. (2018) demonstrated that 3-MA pretreatment reversed the adipose derived mesenchymal stem cells (ADMSCs)-mediated attenuation of chondrocyte apoptosis. In this study, we found that the anti-apoptotic effect of circ_0005567 on IL-1β-induced chondrocytes was abrogated by the autophagy inhibitor 3-MA, indicating that the circ_0005567 inhibited IL-1β-induced chondrocyte apoptosis through inducing autophagy. Our results confirmed the chondroprotective effect of autophagy in OA.

Circular RNAs can function as miRNA sponges or competing endogenous RNAs (ceRNAs) that sequester and competitively inhibit miRNA activity (Liu et al., 2016; Zhou Z. B. et al., 2018; Shen et al., 2019). Using bioinformatics analysis, RNA pull-down, and luciferase reporter assays, we confirmed that circ_0005567 sponged miR-495 to derepress ATG14 expression. ATG14 can activate and initiate the biogenesis of autophagosomes (Obara and Ohsumi, 2011). Our results showed that ATG14 knockdown decreased expression of LC3 puncta, LC3-II, and Beclin-1. Also, ATG4 was identified as a direct target of miR-495, which exerted autophagy-inhibitory effects in IL-1β-treated chondrocytes. More importantly, the autophagy-promoting effect of circ_0005567 overexpression could be abolished by miR-495 mimic and ATG14 knockdown. Hence, we concluded that circ_0005567 promoted autophagy by sponging miR-495 to derepress ATG14 expression. This is the first report demonstrating the involvement of the “circ_0005567-miR-495-ATG14” axis in autophagy regulation. We also found that the anti-apoptotic effects of circ_0005567 overexpression on IL-1β-treated chondrocytes were counteracted by miR-495 mimic and ATG14 knockdown, further suggesting that the “circ_0005567-miR-495-ATG14” axis participates in the pathogenesis of OA.

A previous study showed that circ_0005567 expression was increased under mechanical stress in chondrocytes. Furthermore, circ_0005567 regulated expression of tumor necrosis factor alpha (TNF-α) by sponging miR-875 and participated in the chondrocyte ECM degradation process, which suggested that knockdown of circ_0005567 could be a potential therapeutic target for OA (Liu et al., 2017). This is rather inconsistent with our results. The reason for the contradictory findings is not clear, which requires further investigation.

In summary, this study demonstrated for the first time that circ_0005567 promoted chondrocyte autophagy by sponging miR-495 to derepress ATG14 expression, and thereby inhibited IL-1β-induced chondrocyte apoptosis. The “circ_0005567-miR-495-ATG14” axis could be a promising therapeutic target in autophagy regulation and OA therapy.

## Data Availability Statement

The raw data supporting the conclusions of this article will be made available by the authors, without undue reservation.

## Author Contributions

JZ designed the experiments and wrote the manuscript. All authors performed the experiments, read and approved the final manuscript. JZ and BG analyzed the data.

## Conflict of Interest

The authors declare that the research was conducted in the absence of any commercial or financial relationships that could be construed as a potential conflict of interest.

## Supplementary Material

FIGURE S1 circ_0005567 silencing abrogated the effects of circ_0005567 overexpression on cell apoptosis and ATG14 expression. **(A)** Cell apoptosis rate determined by flow cytometry after Annexin V-FITC/PI staining and **(B)** ATG14 mRNA level determined by qRT-PCR analysis in chondrocytes co-transfected with circ_0005567 overexpression vector/empty vector and si- circ_0005567/si-Ctrl in the presence of IL-1β. The data are expressed as mean ± standard deviation from three independent experiments. **P* < 0.05, ***P* < 0.01, versus the Vector + si-Ctrl group; ^&&^*P* < 0.01, versus the circ_0005567 + si-Ctrl group.

FIGURE S2 3-MA abrogated the circ_0005567 overexpression-mediated promotion of autophagy. The protein levels of Beclin-1 and LC3-II/LC3-I examined by western blot in chondrocytes in the groups of Control, IL-1β, IL-1β + Vector, IL-1β + circ_0005567, IL-1β + circ_0005567 + 3-MA. The data are expressed as mean ± standard deviation from three independent experiments. ***P* < 0.01, versus the Control group; ^##^*P* < 0.01, versus the IL-1β + Vector group; ^&&^*P* < 0.01, versus the IL-1β + circ_0005567 group.
