# Supplementary figures and images for: Hsa_circ_0005567 Activates Autophagy and Suppresses IL-1β-Induced Chondrocyte Apoptosis by Regulating miR-495
Source: Front Mol Biosci. 2020 Aug 25;7:216. doi: 10.3389/fmolb.2020.00216 (PMC7477291; doi:10.3389/fmolb.2020.00216)

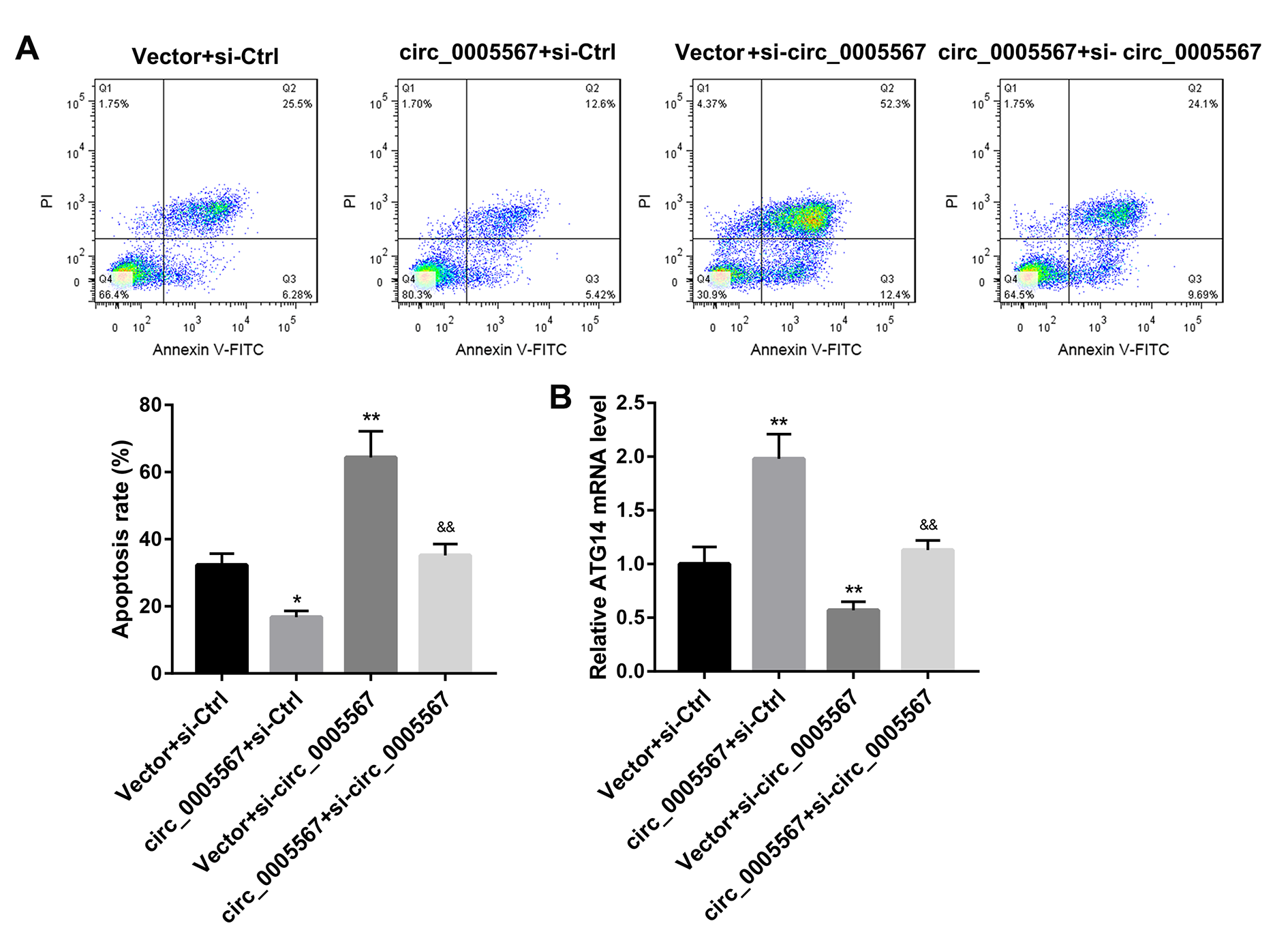

Supplement: FIGURE S1 — circ_0005567 silencing abrogated the effects of circ_0005567 overexpression on cell apoptosis and ATG14 expression. (A) Cell apoptosis rate determined by flow cytometry after Annexin V-FITC/PI staining and (B) ATG14 mRNA level determined by qRT-PCR analysis in chondrocytes co-transfected with circ_0005567 overexpression vector/empty vector and si- circ_0005567/si-Ctrl in the presence of IL-1β. The data are expressed as mean ± standard deviation from three independent experiments. *P < 0.05, **P < 0.01, versus the Vector + si-Ctrl group; &&P < 0.01, versus the circ_0005567 + si-Ctrl group. [file Image_1.tif]

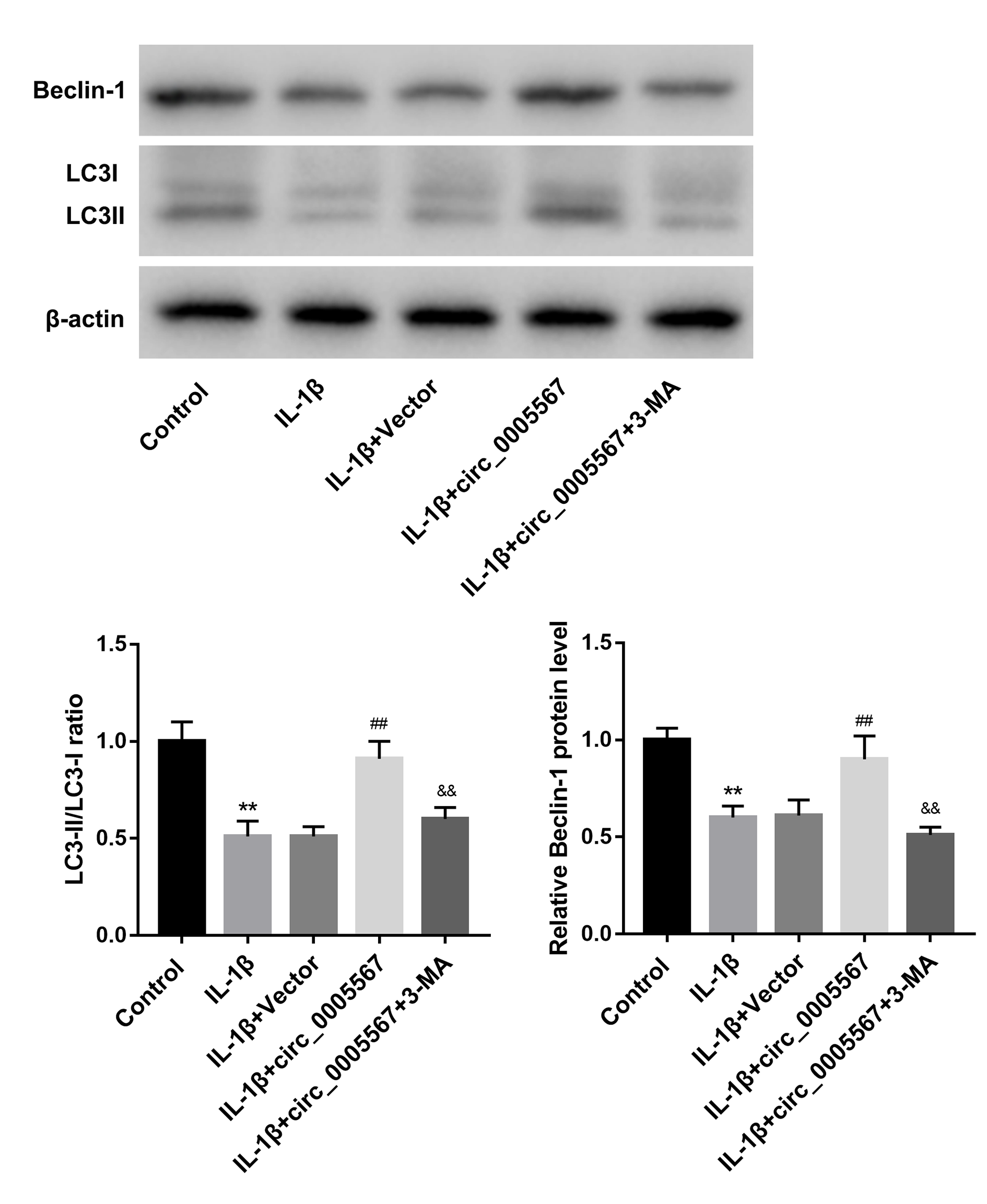

Supplement: FIGURE S2 — 3-MA abrogated the circ_0005567 overexpression-mediated promotion of autophagy. The protein levels of Beclin-1 and LC3-II/LC3-I examined by western blot in chondrocytes in the groups of Control, IL-1β, IL-1β + Vector, IL-1β + circ_0005567, IL-1β + circ_0005567 + 3-MA. The data are expressed as mean ± standard deviation from three independent experiments. **P < 0.01, versus the Control group; ##P < 0.01, versus the IL-1β + Vector group; &&P < 0.01, versus the IL-1β + circ_0005567 group. [file Image_2.TIF]
